# Effects of Subcutaneous Ochratoxin-A Exposure on Immune System of Broiler Chicks

**DOI:** 10.3390/toxins11050264

**Published:** 2019-05-11

**Authors:** Shahzad Akbar Khan, Emerson J. Venancio, Mario A. Ono, Eduardo V. Fernandes, Elisa Y. Hirooka, Cleverson F. Shimizu, Alexandre Oba, Karina K. M. C. Flaiban, Eiko N. Itano

**Affiliations:** 1 Department of Pathologic Sciences, State University Londrina, P.O. Box 10.011, Londrina 86057-970, PR, Brazil; emersonj@uel.br (E.J.V.); marioono@uel.br (M.A.O.); eduardovignoto@uel.br (E.V.F.); 2 Department of Food Science and Technology, State University Londrina, P.O. Box 10.011, Londrina 86057-970, PR, Brazil; elisahirooka@hotmail.com (E.Y.H.); cvsvetuel@gmail.com (C.F.S.); 3 Department of Zootechny, State University Londrina, P.O. Box 10.011, Londrina 86057-970, PR, Brazil; oba@uel.br; 4 Department of Preventive Veterinary Medicine, State University Londrina, P.O. Box 10.011, Londrina 86057-970, PR, Brazil; kkflaiban@uel.br

**Keywords:** leukocytes, lymphoid organs, mycotoxin, chicken immunoglobulins, poultry

## Abstract

Ochratoxin A (OTA), an immunosuppressive mycotoxin, can increase the risk of many infectious diseases and contribute to economic losses to the poultry industry. The immunosuppressive effect has mainly been investigated through oral exposure; however, birds may also be contaminated through skin absorption. The present study investigated the influence of OTA exposure on the defense system of broiler chicks through the subcutaneous route and including low doses. Groups of broiler chicks (Cobb), 05 days old, were exposed to subcutaneous inoculation of OTA at concentrations of 0.1; 0.5; 0.9; 1.3; and 1.7 mg OTA/kg body weight. The size of the lymphoid organs, circulating immune cells, and total IgY and IgA levels were evaluated 21 days post inoculation. Subcutaneous OTA exposure decreased the weight of the thymus, spleen, and bursa of Fabricius, and leukocytopenia (*p* < 0.05) was detected in chicks of the OTA treated groups. In a dose-dependent way, decreased levels of circulating lymphocytes and heterophils (*p* < 0.05), and increased levels of monocytes (*p* < 0.05) were detected. Decreased IgY and IgA serum concentrations were noted in the OTA treated groups (*p* < 0.05). In conclusion, subcutaneous OTA exposure induces immunosuppression even at low levels.

## 1. Introduction

Ochratoxin-A (OTA), a toxin of fungus produced by some members of *Aspergillus* and *Penicillium* [1,2], was isolated for the first time from *Aspergillus ochraceus* [2,3]. As a natural food contaminant in a wide variety of poultry feeds [4,5], consumption of OTA can cause various deleterious effects. OTA has been described as a mutagenic, teratogenic, nephrotoxic, neurotoxic, hepatotoxic, hematotoxic, and immunotoxic mycotoxin [6,7,8,9,10,11].

Regarding immunosuppression, OTA suppresses the immune system of chickens, causing atrophy and a decrease in overall weights of the immunological organs including both primary lymphoid organs, the thymus and bursa of Fabricius, and peripheral lymphoid organs such as the spleen [7]. The central lymphoid organs are lymphocyte producing organs [12] and severe lymphocytopenia, as well as depression of antibody response, have been observed in poultry exposed to OTA [6,13,14], in high [6,13,14] or low doses [15,16], such as the optimum daily value of OTA for poultry feed recommended by the European Commission Recommendation 2006/576/EC [17].

Ochratoxin A immunotoxicity can increase the risk of infectious diseases and accounts for economic loss to the poultry sector. Although the oral route of OTA exposure is frequently employed in studies to evaluate the effects on the immune system, this is not an exclusive route of contamination; humans and animals may also be contaminated through skin absorption [18,19]. To date, there are no data on birds, but an in vitro human skin model demonstrated higher diffusion of OTA compared to other mycotoxins (AFB1, FB1, CIT, ZEA, and T-2) [20].

Taking into account that no previous study in poultry is available on the influence of subcutaneous Ochratoxin A exposure on the defense system of broiler chicks, this study introduced a subcutaneous route of OTA exposure, including the dose recommended by the EU for poultry feeds (0.1 mg/kg feed) [17] and higher OTA doses.

## 2. Results

### 2.1. Relative Weights of the Bursa, Thymus, and Spleen

**Figure 1:**
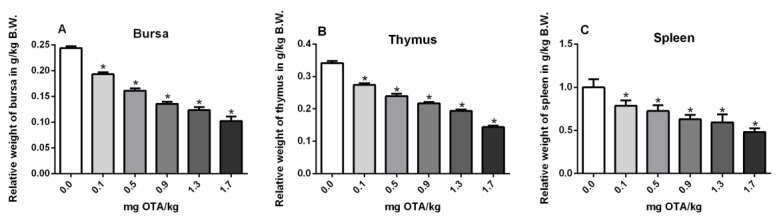
Relative weights of the bursa (**A**), thymus (**B**), and spleen (**C**) in OTA-treated or not treated chicks. n = 6 (for each group). Analysis performed through the one-way ANOVA test followed by the Bonferroni post hoc. Data presented as mean ± sd. *, Significant in comparison to control not treated with OTA (*p* < 0.05). B.W.: Body Weight.

The corresponding overall weights of the bursa (Figure 1A), thymus (Figure 1B), and spleen (Figure 1C) were significantly decreased at all levels of OTA exposure by the subcutaneous route in chicks at 21 days p.i., in relation to the control group (Figure 1A–C) (*p* < 0.05; Figure 1).

### 2.2. Circulating Leukocytes and Differential Counts

**Figure 2:**
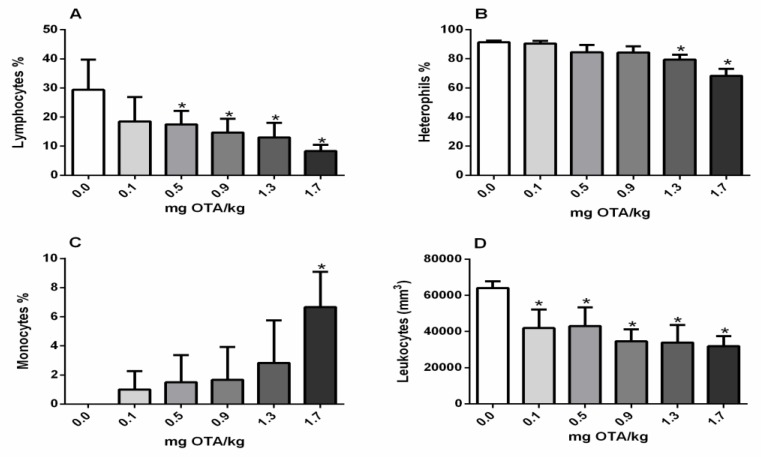
Effects of OTA exposure on circulating leukocytes (cells/mm^3^) and differential (%) counts in broiler chicks. (**A**) lymphocytes (%); (**B**) heterophils (%), (**C**) monocytes (%), and total circulating leukocytes (cells/mm^3^) (**D**). n = 6 (for each group). Analysis performed through the one-way ANOVA test followed by the Bonferroni post hoc. Data presented as mean ± sd. *, Significant in comparison to control not treated with OTA (*p* < 0.05).

Total leukocytes were significantly decreased in the experimental chicks exposed through the subcutaneous route at every dose level of OTA, in relation to the non-treated group (*p* < 0.05; Figure 2D). The level of lymphocytes decreased after exposure to doses of 0.5mg and higher of OTA (Figure 2A) and heterophils reduced at doses of 1.3 and 1.7 mg of OTA/kg B.W. (Figure 2B). On the other hand, monocyte count was higher in chicks treated with 1.7 mg OTA/kg B.W. (Figure 2D).

### 2.3. Serum Values of Total IgY and IgA in Chicks

**Figure 3:**
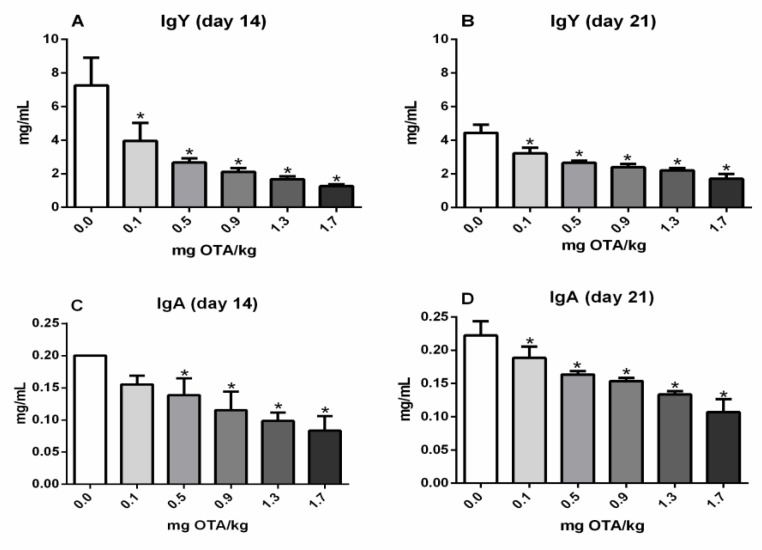
Total IgY and IgA in the serum of exposed chicks (OTA) by immunocapture ELISA. (**A**) IgY 14 days post s.c. inoculation, (**B**) IgY 21 days post s.c. inoculation, (**C**) IgA 14 days post s.c. inoculation, and (**D**) IgA 21 days post s.c. inoculation with 0.0, 0.1, 0.5, 0.9, 1.3, and 1.7 mg OTA/kg b.w. n = 6 (for each group). Analysis performed through the one-way ANOVA test followed by the Bonferroni post hoc. Data presented as mean ± sd. *, a significant decrease in comparison to control not treated with OTA (*p* < 0.05).

The total IgY serum values were reduced in all groups treated with Ochratoxin-A compared with the non-treated control at day 14 (*p* < 0.05; Figure 3A) and 21 (*p* < 0.05; Figure 3B). Moreover, the total IgA serum values were significantly decreased in the experimental groups of chicks inoculated with 0.5, 0.9, 1.3 mg, and 1.7 mg OTA/kg 14 days post inoculation (p.i.) (*p* < 0.05; Figure 3C). At 21 days p.i., similarly, the IgY and IgA values were significantly reduced in all groups treated with OTA (*p* < 0.05; Figure 3D).

### 2.4. Total Serum Protein Levels in Chicks Treated with Ochratoxin A

**Figure 4:**
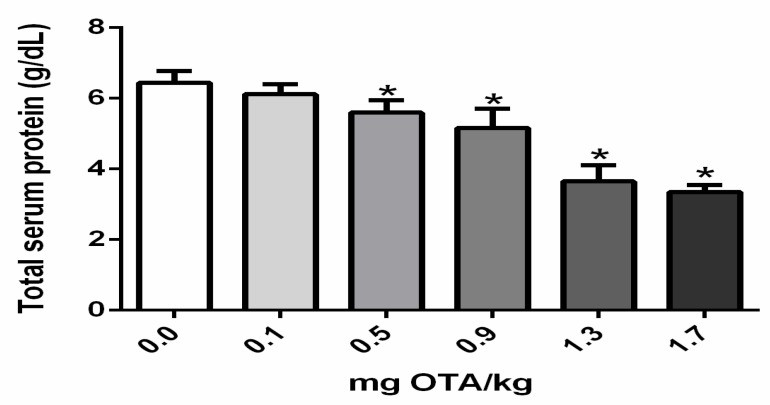
Total serum protein levels in chicks treated with Ochratoxin-A. Protein values are represented in g/dL; n = 6 (for each group). Analysis performed through the one-way ANOVA test followed by the Bonferroni post hoc. Data presented as mean ± sd. *, a significant decrease in comparison to control not treated with Ochratoxin-A (*p* < 0.05).

Total serum protein level significantly declined in chicks exposed to 0.5, 0.7, 0.9, 1.3, and 1.7 mg OTA/kg B.W. in comparison to the control not treated with OTA (*p* < 0.05; Figure 4).

## 3. Discussion

The presence of mycotoxins or fungal conidia in airborne dust, often at high levels, has been evidenced in different workplaces, such as agricultural and food processing environments, as well as in water-damaged environments [21,22,23]. In these areas, mycotoxins can contaminate animals or humans through inhalation or skin absorption [22,23]. Although the skin absorption route is an important route of low molecular weight chemicals [24], it is often overlooked in relation to mycotoxins, which also present small molecular weight [19]. Mycotoxins are able to penetrate through human skin [18,20,25] and OTA is a mycotoxin that demonstrates high ability to diffuse through human skin [20].

Herein, chicks exposed to OTA through the subcutaneous route demonstrated a reduction in the bursa and thymus similar to that previously observed by the oral route at the lowest OTA dose (0.1 mg/kg feed) [16], but differently, the subcutaneous route also induced a reduction in spleen size at the lowest OTA dose (0.1 mg/kg B.W.). As the oral contamination was performed on day 1 [16] and the subcutaneous contamination on day 5, the immune system of the chicks was already more developed. Despite this, the spleen, a peripheral organ, was affected by a lower dose, suggesting greater sensitivity of this organ when OTA exposure occurs through a subcutaneous route. This higher sensitivity may be due to the higher concentration of OTA in lymphoid organs when exposed subcutaneously. It is already well known that lymphatic vessels contain structures that differ from blood vessels, allowing easy absorption of substances found in tissues, which are transported to the secondary lymphoid organs [26]. Therefore, OTA injected subcutaneously may be absorbed by the lymphatic vessels and transported to peripheral lymphatic organs such as the spleen. On the other hand, exposure to OTA through feed must pass through the gastrointestinal tract. Especially in birds, the percentage of absorption is very low in relation to other species [27]. The low OTA uptake by this species may be due to longer intestinal retention time and degradation by intestinal microorganisms that hydrolyze OTA [28]. After absorption by the gastrointestinal tract, the OTA could be distributed mainly via the hematogenic route. It has been demonstrated, in rats, that the initial migration route of the toxin is through the portal vein. The increased ratio of detection of the toxin in plasma in comparison to lymph might be due to the higher passing rate of blood in comparison to lymph [29]. Mice exposed to T-2 toxin either by dermal or subcutaneous routes caused significant oxidative damage in the brain [30], suggesting absorption and possible systemic diffusion to reach the brain. There is a large gap in relation to OTA and birds in this sense.

Possibly as a consequence of a decrease in the size of the bursa and thymus, central lymphoid organs that are responsible for lymphocytes production [12], similarly to that previously observed for oral exposure [16], presented a decreased percentage of circulating lymphocytes by the subcutaneous route. The majority of previous studies concerning lymphopenia have investigated the result of OTA through oral exposure and also with higher doses of OTA [13,31]. Similarly to that observed for exposure to OTA via feed [15], there was an increase in the percentage of monocytes at high levels of exposure. However, monocytopenia has been described in the literature [13], which could be due to age and genetic diversity and requires further study.

Interestingly, although heterophils increased after oral exposure [16,32] they were significantly reduced in groups exposed to OTA subcutaneously. Heterophils, like mammalian neutrophils, are phagocytic cells and form the first line of natural defense against invading microbial pathogens [33]. This subcutaneous route is presumably more deleterious for chicks, by reducing the heterophils and also decreasing total circulating leukocytes. Leukocytopenia has been described frequently with high doses of OTA [13,34], including previous oral data with the dose of 0.3 mg/kg feed [16]; however, by the subcutaneous route leukocytopenia was detected even at a low dose.

Decreased specific antibody responses following OTA exposure have been noted in chickens, although, frequently, at higher doses of OTA [6,14,35,36]. In the present study, as expected, total IgY and IgA levels were also reduced in all groups of animals treated with OTA subcutaneously, similar to previous data obtained on oral exposure with low doses of OTA, at 21 days p.i. [16]. Antibody levels may be reduced due to a quantitative decrease in lymphocytes, especially antibody-producing lymphocytes. In this work, only a reduction in total lymphocytes was evidenced and the antibody-producing B lymphocytes were not investigated. Additionally, antibodies, as proteins, can also be reduced due to inhibition of synthesis of protein. The stoppage of peptide elongation via competition with Phe-tRNA synthetase is the most common way of stoppage of synthesis of protein by Ochratoxin A [37]. OTA also acts as a regulator of intracellular transcription of many proteins, which may interfere with protein synthesis [38].

To our knowledge, this is the first investigation on the influence of Ochratoxin-A on the immune system of chicks exposed subcutaneously. In the overall context, the s.c. route of OTA exposure demonstrated a greater effect on the immune system, such as the reduction in the secondary lymphoid organ (spleen), at a low dose (s.c. 0.1 mg OTA/kg B.W. versus oral 0.9 mg OTA/kg feed), a decrease in circulating leukocyte with the lowest dose (s.c. 0.1 mg OTA/kg B.W. versus oral 0.3 mg OTA/kg feed), and a decrease in heterophils (s.c. decrease 1.3 mg OTA/kg B.W. versus increase in oral 0.3 mg OTA/kg feed). In addition, at 14 days post exposure, the IgA level was reduced at a lower dose compared to oral administration (s.c. 0.5 mg OTA/kg B.W. versus oral 0.7 mg OTA/kg feed). As previously discussed, similarities of effects between the two routes of exposure were also observed, such as a reduction in primary lymphoid organ sizes (s.c. 0.1 mg OTA/kg B.W. or oral 0.1 mg OTA/kg feed), a reduction in lymphocytes (s.c. 0.5 mg OTA/kg B.W. or oral 0.5 mg OTA/kg feed), a decrease in total circulating protein levels (s.c. 0.5 mg OTA/kg B.W. or oral 0.5 mg OTA/kg feed), and a reduction in IgY antibody levels (s.c. 0.1 mg OTA/kg B.W. or oral 0.1 mg OTA/kg feed).

In contaminated environments, dust particles containing mycotoxins can be deposited on the skin or on other surfaces that can be touched and create opportunities for dermal absorption through human occupational exposure [39]. In addition, chickens present the habit of spraying food or lying on the ground, which in contaminated environments would make them likely to come into contact with mycotoxins via the skin.

As discussed previously, subcutaneous and also dermal exposure could be collected mainly by lymphatic vessels [26]. Through lymphatic circulation, OTA can quickly reach peripheral lymphoid organs or nodes and cause damage; additional studies are required to investigate this pathway.

By oral exposure, OTA reaches the bloodstream after absorption by the gastrointestinal tract. Within the bloodstream, OTA could bind to circulating proteins [40]. According to the literature, OTA exhibits high affinity for serum albumin and other macromolecules in the blood [41,42]. OTA bound to proteins seems to remain for a longer period in the bloodstream and present an impact on the biological half-life of OTA [40,41,42,43]. Future investigations should be carried out with subcutaneous exposure to OTA at doses lower than 0.1 mg/kg B.W., and including the OTA dosage in the blood and in the lymphoid and non-lymphoid organs.

## 4. Conclusions

Subcutaneous OTA exposure induces suppressive effects at low doses on the immune system of chicks, possibly with greater impact than observed in the literature data on oral exposure.

## 5. Materials and Methods

### 5.1. Experimental Chicks and Their Management

The present study was conducted with 36, 5-day-old, specific pathogen-free (SPF) experimental chicks (Cobb) from a hatchery located in Londrina, Parana, Brazil, for a duration of 3 weeks. The chicks procured for the study were from the same parent stock. Before the introduction of the chicks, the poultry houses were washed with clear water and then disinfected with KMnO4 and formalin (1:2). The chicks were fed with broiler mash feed and a standard environment was provided for the whole period of the study. Before the start of the experiment, all feed was checked for the presence or absence of mycotoxins, and all the feed material was found to be free from mycotoxins. Feed [44] and water were provided ad libitum throughout the study. The chicks did not receive any hormones, growth promoters, or antibiotics throughout the study.

### 5.2. OTA-Dose Preparation

OTA (CAYM 11439; Cayman Chemical Company, Ann Arbor, MI, USA) was resuspended in ethanol (1 mg OTA/10 mL ethanol) in order to dissolve it completely. This suspension was then uniformly mixed and different doses were adjusted for subcutaneous inoculation according to pre-determined levels.

### 5.3. Experimental Design

On day 1, 36 chicks were divided into six groups, six birds in each group and then acclimatized for 5 days. At 5 days of age, five groups were inoculated subcutaneously with OTA at doses of 0.1, 0.5, 0.9, 1.3, and 1.7 mg OTA/kg of body weight, respectively. One group not exposed to OTA served as a control. All experiments were approved by the Animal Care and Ethics Committee on the use of animals, State University of Londrina, PR, Brazil (CEUA). (identification code: CEUA 18419.2013.89; date of approval: 29 November 2013).

### 5.4. Relative Weights of the Bursa, Thymus, and Spleen

On termination of the experiment, all the animals were euthanized by the Halal method. All lymphoid organs (bursa of Fabricius, the thymus, and the spleen) were weighed properly in a separate way and correlations with the total body weight were calculated.

### 5.5. Circulating Leukocyte Profiles and Differential Counts

Blood was collected for hematological analysis on termination of the experiment. Approximately 2 mL of blood was collected from the brachial vein of each broiler in 5% ethylenediaminetetraacetic acid. Total leukocyte concentrations were obtained using a 1:200 dilution with Natt Herrick solution by counting all the leucocytes in the nine large squares in the ruled area of the hemacytometer chamber using the formula (TWBC/mm^3^ = total cells in nine large squares + 10% × 200) after staining for 60 min. The differentiation of leukocytes proceed in the blood film (manual counting by using optical microscopy).

### 5.6. Quantification of IgY and IgA

Two mL of blood was collected from the brachial vein at 14 and 21 days post-Ochratoxin-A treatment. Blood was allowed to clot and serum samples were obtained after centrifugation and were stored at (−20 °C) until use. Immunocapture ELISA was performed for determination of IgY and IgA concentrations in the serum of all control and experimental groups using commercial kits (Bethyl Laboratories, Montgomery, TX, USA). Serum was diluted at 1:200,000 for IgY and 1:500 for IgA. Levels of IgY and IgA were calculated based on standard curves.

### 5.7. Total Serum Protein Levels in Chicks Treated with OTA

Serum samples were separated from the blood of each representative chick and were used to evaluate the levels of serum total protein. The total protein concentration was determined in a biochemical autoanalyser (Dimension Xpand Plus, Siemens, Berlin, Germany) by colorimetric method using commercial reagents (Total Protein Flex, Siemens). Results were expressed in g/dL.

### 5.8. Statistical Analysis

All data were subjected to homogeneity (Levine’s test) and normality (Kolmogorov test). Data were analyzed by one-way analysis of variance. Means of all the groups were compared by Bonferroni tests using GraphPad Prism statistical package 5.01. Data were considered significantly different when *p* values were less than 0.05.
